# Multidrug-Resistant Hepatitis B Virus Strain in a Chronic Turkish Patient

**Published:** 2010-06-01

**Authors:** Murat Sayan, Sadettin Hulagu, Sinem Ceren Karatayli

**Affiliations:** 1 Clinical Laboratory, Faculty of Medicine, University of Kocaeli, Kocaeli, Turkey; 2 Department of Gastroenterology, Faculty of Medicine, University of Kocaeli, Kocaeli, Turkey; 3 Institute of Hepatology, University of Ankara, Ankara, Turkey

**Keywords:** Hepatitis B Virus, Multidrug-Resistant, Nucleoside/Nucleotide Analogues, Direct Sequencing, Line Probe Assay, Clonal Analysis

## Abstract

**Abstract:**

Hepatitis B virus (HBV) strains, resistant to at least two anti-HBV agents from different subclasses of nucleos(t)ide analogues (NUCs) without a cross-resistance profile, are defined as multidrug-resistant. However, there are limited in vivo data for resistance to multiple NUCs. In this study, we report the case of the emergence of a multidrug-resistant HBV strain in a Turkish patient receiving sequential therapy. Polymerase gene mutations of HBV were detected using direct sequencing, line probe assay and clonal analysis. Twelve months after the start of lamivudine (LAM) therapy, virological breakthrough occurred (4.2E+07 IU/ml) and the rtM204V variant was detected in the patient’s sera: adefovir (ADV) was added to the therapy. ADV therapy was continued as monotherapy for 11 months, until the occurrence of clinical breakthrough i.e. alanine aminotransferase (ALT) 60 IU/L, and emergence of drug resistance to ADV (rtN236T). At that time, switch therapy was resumed with ADV + entecavir (ETV) in combination for 5 months. In the 18th month of the ETV monotherapy, direct sequencing showed reduced susceptibility to ETV (rtL180M+rtM204V). Currently, ETV + tenofovir (TDF) are being used as antiviral treatment and the HBV DNA load has decreased substantially (<1.0E+02 IU/ml). In conclusion, we have detected an HBV strain with multidrug-resistance, which had a very fast course of development. Patients with a multidrug-resistant profile should be more frequently followed up both by direct sequencing and line probe assay, for the detection of possible novel HBV variants and low level mutants present in the viral population, in case of the sudden emergence of drug resistance.

## Introduction

Two different types of drugs can be used in the treatment of chronic hepatitis B (CHB): interferon alpha and nucleos(t)ide analogues (NUCs). NUCs for hepatitis B virus (HBV) therapy belong to three subclasses: L-nucleosides (lamivudine [LAM], telbivudine [LdT], and emtricitabine), deoxyguanosine analogues (entecavir [ETV]) and acyclic nucleoside phosphonates (adefovir [ADV] and tenofovir [TDF]). LAM, LdT, ETV, ADV, and TDF have been approved in Europe, the United States, and most Asian and Latin American countries, for HBV treatment [1][2][3].

A major concern with NUC treatment is the selection of antiviral – resistant mutations. Long term therapy with NUCs, in particular, is associated with an increasing risk of the development of drug resistance [4][5]. Mutations selected under NUCs can be split into two groups: those that cause resistance that sometimes leads to decreased viral fitness, and compensatory mutations, which partially or fully restore the level of viral fitness [6][7]. HBV strains resistant to at least two anti-HBV agents from different subclasses of NUCs without a cross-resistance profile are defined as multidrug-resistant [3]. The multidrug-resistant strains are more likely to develop in sequential therapy [8][9]. The emergence of a multidrug-resistant HBV strain under sequential oral anti-HBV therapy has recently been documented for the first time [10]. Nevertheless, there are limited in vivo data for resistance to multiple NUCs [3].

Turkey, with about 6500 individuals newly infected with HBV a year, is numbered among those regions with intermediate endemicity [11][12][13]. Moreover, a few studies of Turkish patients with treated or untreated CHB infections have frequently indicated HBV drug resistance as rtM204V (YVDD variant), rtM204I (YIDD variant) and, rarely, as rtM204S (YSDD variant) mutations with or without compensatory mutations, such as rtV173L and rtL180M [14][15][16][17]. Although little is known about multidrug-resistant HBV strains, a recently reported novel mutation pattern emerging during LAM treatment shows cross - resistance to ADV treatment [9].

In this study, we report the case of a Turkish patient who had clinical and genotypical breakthrough under NUC treatment, and who was shown to be multidrug-resistant to sequential therapy.

## Case Report

**Figure 1:**
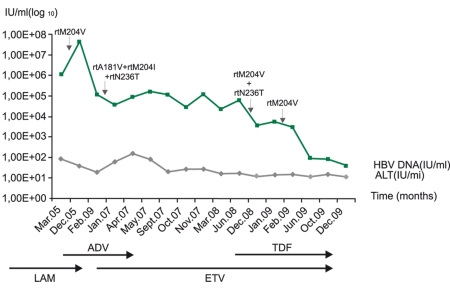
Obtained sequential data on the HBV-DNA loads and ALT levels with primer drug resistance during sequential nucleos(t)ide analogues therapy.

The patient was a 33-year-old female with hepatitis B e antigen (HBeAg)-negative CHB. A liver biopsy in 2004 revealed CHB and the patient was scored as grade II and stage II. Liver damage was determined according to Knodell’s classification [18]. Oral antiviral therapy was started with LAM (Zeffix100-® mg/day, Glaxo Wellcome Laboratories, Middlesex, UK). However, 12 months after the start of LAM therapy, virological breakthrough occurred (HBV DNA 4.2E+07 IU/ml) and the YVDD variant was detected. ADV (Hepsera-®10 mg/day, Gilead Sciences, Inc. Foster City, CA 94404. U.S.A) was added to LAM therapy in the 13th month of antiviral treatment. After two months, ALT normalization and a decrease in the HBV viral load were observed. ADV therapy was continued as monotherapy for 11 months. After the occurrence of clinical breakthrough i.e. alanine aminotransferase (ALT) 60 IU/L, and the emergence of drug resistance to ADV, switch therapy was agreed on, and antiviral treatment was resumed with ADV + ETV (Baraclude1 -® mg/day, Bristol-Myers Squibb Company Princeton, NJ U.S.A) in combination for 5 months, followed by ETV monotherapy for 18 months. In the 18th month of ETV monotherapy, ALT was at a normal level, and the HBV viral load dropped to 3.54E+03 IU/ml. Direct sequencing, however, showed reduced susceptibilty (intermediate) to ETV. After the occurrence of resistance to ETV, TDF was added on to the ETV monotherapy as the current treatment regimen, and ETV + TDF (Viread® -245 mg/day, Gilead Sciences, Inc. Foster City, CA U.S.A) therapy decreased the HBV DNA load (2.84E+02 IU/ml). Currently,the latest HBV DNA levels of the patient over the last 6 months are <100 IU/ml. Sequential NUC therapies and developing primer / compensatory resistance detected by direct sequencing, line probe assays (LIPA) and clonal analysis of the HBV variants (in 2 different sera samples – taken at the 11th month of ADV therapy and at the 4th month of ETV + TDF therapy) are summarized in Table 1.HBV DNA load status and ALT levels with primer drug resistance are plotted in Figure1.

### Hepatitis serology and HBV DNA assays

Hepatitis B surface antigens (HBsAg), anti-HBsAg antibodies (anti-HBs), anti-HBeAg antibodies (anti-HBeAg) and anti-hepatitis C virus antibodies (anti-HCV) were determined by the microparticle enzyme-immunoassay method and anti-D virus antibodies (anti-HDV) by an enzyme-immunoassay (Abbott Laboratories, IL,USA). HBV DNA levels were tested using a commercial real- time polymerase chain reaction (real time PCR) (Iontek Biotechnology Inc, Istanbul, Turkey). A nested polymerase chain reaction (PCR) for HBV was used, as previously reported by Karatayli et al.[9].

### The sequencing of the HBV polymerase gene region

Briefly, a pair of primers was designed (forward: 5’-TCGTGGTGGACTTCTCTCAATT-3’ and reverse: 5’- CGTTGACAGACTTTCCAATCAAT-3’) for amplification of the HBV polymerase region. The PCR conditions were: 95ºC for 15 min, and then 45 cycles consisting of 95ºC for 45 s, 56ºC for 45 s, and 72ºC for 45 s. The final concentration of the primers was 0.3 µM. The size of the HBV amplicon was 742-bp. All PCR products were purified using the High Pure PCR Product Purification Kit® (Roche Diagnostics GmbH, Mannheim, Germany) and directly sequenced with ABI PRISM 310 Genetic Analyzer® equipment, using the DYEnamic ET Terminator Cycle Sequencing Kit® (Amersham Pharmacia Biotech Inc, Piscataway, NJ USA). For the cycle sequencing, the following thermal protocol was used: 35 cycles consisting of 95ºC for 20 s, 50ºC for 25 s, and finally 60ºC for 2 min. The reverse primer was used as the sequencing primer at a final concentration of 0.5 uM. The electropherogram-obtained sequences were assembled using Vector NTI® v5.1 (InforMax(TM) Invitrogen(TM) life science software, Frederick, MD 21704, USA).

### Determination of HBV genotypes

HBV genotypes were determined by means of the genotyping tool of the National Center for Biotechnology Information (NCBI, U.S National Library of Medicine, Bethesda, MD USA, http://www.ncbi.nih.gov/projects/genotyping/formpage.cgi.), which identifies the genotype, based on viral nucleotide sequences. The genotyping tool works by using BLAST to compare a query sequence to a set of reference sequences for known genotypes [19].

### Determination of polymerase gene mutations

Polymerase gene mutations of HBV were detected by manual analysis, a drug resistance tool and LIPA. The Genafor/Arevir - geno2pheno drug resistance tool (Center of Advanced European Studies and Research, Bonn,Germany, http://coreceptor.bioinf.mpi-inf.mpg.de/) for HBV is a database that is specifically designed for rapid computer - assisted virtual phenotyping of HBV, and accepts genome (nucleic acid) sequences as input. Geno2pheno searches for homology between input sequences and others already stored in its database, which also stores relevant clinical data for drug resistance and surface (S) gene mutations. The data accumulated by direct sequencing were analyzed either manually or by using the geno2pheno tool. The geno2pheno tool searches for HBV drug resistance mutations in the rt amino acids at positions 80, 169, 173, 180, 181, 184, 194, 202, 204, 215, 233, 236, and 250. However, the reverse transcriptase (rt) domain of the polymerase at amino acid positions 84, 85, 214, 237, and 238 was additionally searched for, manually [4][20]. The Inno-LIPA HBV DR v2 assay (Inno-LIPA; Innogenetics NV, Ghent, Belgium) was performed according to the manufacturer’s instructions using the QuantiTect SYBR Green PCR kit (Qiagen, Hilden,Germany). The assay is based on the amplification of a part of the viral polymerase gene by the provided primers and reverse hybridization by probes coated on a strip. It detects HBV polymerase wild-type and known drug-induced mutations associated with LAM and ADV resistance (codons 80, 173, 180/181, 204, and 236). The patient was infected with genotype D of HBV and the data, obtained from positions 80 through 250 of the amino acids of the HBV polymerase gene by direct sequencing, were analyzed either manually or by using the geno2pheno tool. The results were similar in both cases.

### Clonal Analysis of HBV variants

Clonal analysis has been performed on 2 different sera samples obtained and also on 3 different extraction samples obtained during the patient’s therapy. However, the cloning procedure was successful in only 2 sera samples, and clones obtained from these sera samples have been sequenced. For the cloning of the HBV polymerase region, a 700 bp PCR amplification covering the whole HBV pol gene was done, using the following primers; CLC188 5’-TCCCCAACCTCCAATCAC-3’ and CLC887 5’-AAACCCAAAAGACCCACAA-3’. The PCR was run for 35 cycles, with denaturation at 95 °C for 45 s, annealing at 55 °C for 30 s, and elongation at 72 °C for 1 min. The amplified 700 bp HBV polymerase region was cloned into a TA vector by using the TOPO-XL-PCR Cloning kit (Invitrogen Corp. Carlsbad, CA USA) and the constructs were then sequenced using the Big Dye Terminator v3.1 Cycle Sequencing Kit (Applied Biosystems, Foster City, CA, USA) in an ABI Prism 3130 XL Genetic Analyser (Perkin Elmer, Foster City, CA, USA) according to the manufacturer’s instructions.

## Discussion

Towards the end of 2003, a case report of a patient with HBV infection and antiviral resistance to ADV, after failure of initial therapy with LAM, heralded the new era of HBV multidrug-resistance [21]. Actually, the emergence of HBV variants should have been expected due to the characteristics of the HBV genome. The major causes of drug resistance include viral factors. such as the kinetics of viral production and clearance; lack of a proofreading mechanism during reverse transcription, which creates a large HBV quasispecies pool; and the replication fitness of the viral quasispecies [22]. The emergence of multidrug-resistant strains does not seem to be a common phenomenon [23]. There have been several studies testing HBV mutants for a multidrug-resistance profile to different antiviral drugs [10][24]. A new mutation pattern, rt181S + rtM204I, which arose under LAM treatment, conferring cross-resistance to ADV treatment, has recently been reported in a Turkish patient [9]. Thus, the presence of multidrug-resistant HBV strains is still a major therapeutic problem.

This study has demonstrated that the occurrence of an HBV strain with multidrug-resistant variants, is associated with primer LAM and primer ADV resistance, and with reduced susceptibility to ETV and TDF, according to the new EASL Clinical Practice Guidelines [1]. Three primer drug resistance mutations in different domains of HBV viral polymerase, the rtA181V/T, rtL180M+ rtM204V mutations and the rtN236T mutation, were characterized in the same genome, which might explain the multidrug-resistance profile. The resistance to LAM and ADV of an engineered laboratory mutant HBV strain harbouring the rtL180M + rtM204V+ rtN236T mutations pattern has been described in vitro, and it was suggested that non-optimal strategies based on the sequential use of NUCs might result in the emergence of selected multidrug-resistant strains [25]. In our patient, we applied the “add-on” strategy between sequential monotherapies. The most effective treatment of multidrug resistance is its prevention, by avoiding the sequential use of NUCs monotherapies. However, the choice of the initial treatment strategy may affect the efficacy of future treatment options [23][26]. Moreover, for the avoidance of the emergence of multidrug-resistance, immune control of HBV would probably be required, in addition to oral anti-HBV agents in treating chronic hepatitis infection [3]. On the other hand, drug resistance to LAM developed after 12 months of initial therapy, and to ADV after 13 months of LAM + ADV combination therapy in our case. Those were resistances that developed very suddenly.The cumulative incidence of HBV resistance to LAM and ADV in published pivotal trials in NUC-naive patients in year 1 was 24% and 0%, respectively [1].

The rtM204V mutation in our case was detected as a primer drug resistance to LAM in all applied direct sequencing assays and reconfirmed by LIPA findings. But, the rtN236T mutation responsible for primer ADV drug resistance (besides rtA181V/T) found by LIPAwas never detected in direct sequencing. However, clonal analysis of the HBV variants revealed that the rtM204V + rtV173L + rtL180M mutations pattern was present in all clones in the sera sample of the 4th month of ETV + TDF therapy Table 2. Hence, all of the methods applied in this study are sufficient to establish that the rtM204V + rtV173L + rtL180M mutations are in the same genome and predominate. Detected rtM204I and rtA181V mutations in clones 2, 5 and 4, however, in the other serum sample clonally analyzed, was not consistent with the results of direct sequencing and LIPA assay (Table 1). This might have been due to the low level of these mutations in the HBV quasispecies pool, undetectable by sequencing and LIPA assay. Interestingly, in addition to known primary and compensatory mutations, some previously undocumented mutations, such as R120K, H126R, S135Y, and H248N, were detected in 2 of the 5 clones of serum samples from the 11th month of ADV treatment, and in all clones of serum samples from the 4th month of ETV+TDF treatment. On the other hand, compensatory mutations such as rtV173L, rtQ215H, and rtL80I/V have been detected only by direct sequencing and LIPA, respectively. However, only the rtL180M mutation was detected by both methods. Most of the clones from the sera samples taken in the 11th month of ADV therapy revealed the presence of the rtL80V mutation in the clonal analysis of the HBV variants. These findings suggested that all methods used in this study for resistance detection of HBV variants had different sensitivities and accordingly, both methods should be used on follow-up of the infected case with multidrug- resistant strains, for effective treatment management. NUC resistance in HBV variants is commonly detected by the direct sequencing of HBV DNA [27]. However, this assay is time-consuming for a large number of clinical samples, but is suitable for large-region screening, in viral genomes. LIPA technology has been developed for more sensitive detection of resistance mutation in the HBV polymerase gene. This technology is useful for the rapid and accurate detection of mutants, which make up as little as 5%-10% of the HBV population, but it only detects known mutations for LAM and ADV, together with wild type variants [28][29]. In our study, applied LIPA assays showed wild types for the N236 and M204 amino acid positions, and this finding suggests that wild-type virus strains of rtN236T and rtM204V variants may still be reproducible in this patient’s sera. Also, this possibility may be also specified for compensatory mutations (L80,V173, L180/A181). In this study, amino acids in positions 80 through 250 of the HBV polymerase gene have been manually analyzed and the results were similar to those from the geno2pheno tool analysis. Databases, such as the geno2pheno tool are a convenient approach in HBV mutation phenotyping. Large regions of sequence data can be rapidly interpreted for amino acid substitutions with the help of this database. Some compensatory mutations demonstrated in this study (rtL80I/V, rtV173L, rtL180M, rtQ215H) help to restore the replication efficiency of the mutant virus, but they tend to occur in the presence of antiviral selection pressure [30]. The viral load may be an indirect marker of the replication of efficiency. There was no significant relationship between the sequential HBV DNA loads of the patient with the compensatory mutations in the present study. Actually,there has also been no significant relationship between primer drug resistance mutations (the YVDD variant and the rtN236T variant), and HBV DNA loads and ALT levels. (Fig.1). The replication capacity of the detected multidrug-resistant strain requires further investigation. In conclusion, we have detected an HBV strain with multidrug-resistance associated with primer LAM and primer ADV resistance and reduced susceptibility to ETV and TDF. There has not been much experience with multidrug-resistance in CHB patients. Patients with multidrug-resistance could be more frequently followed up by direct sequencing and LIPA, for the emergence of drug resistance in possible new and/or low level variants of HBV.

**Table1:** Genotypic resistance patterns of multidrug-resistant HBV strain in sequential NUCs mono and combine therapy.

**Sequential NUCs therapy (Dec. 2004 – Feb. 2009)**	**Resistance detection method (application time in the treatment)**	**Primary resistance**	**Compensatory resistance**	**Resistance to**
LAM	Direct sequencing (13th month)	M552V^a^ (rtM204V)	L528M^a^ (rtL180M), V555V (rtV207V)	LAM and ETV (intermediate)^b^
LAM + ADV	NT^c^	NT	NT	-
ADV	LIPA (11th month)	rtN236T (Wild type:N236)	(Wild type:L80)	ADV and TDF (intermediate)^d^
ADV	Clonal analysis (on 6 clones) (11th month)	rtM204I (in clone 2, 5) rtA181V (in clone 4)	rtL80V (in clone 2, 3, 5)	LAM, LdT, ADV
ADV + ETV	Direct sequencing (5th month)	-	rtQ215H	-
ETV	Direct sequencing (18th month)	rtM204V	rtV173L, rtL180M	LAM and ETV (intermediate)
ETV	LIPA (18th month)	rtM204V, rtN236T, tA181V/T (Wild type:M204, N236)^f^	rtV173L, rtL180M^e^, rtL80I/V (Wild type:L80, V173, L180/A181)^f^	LAM, ADV and TDF (intermediate)
ETV + TDF	Direct sequencing (4th month)	rtM204V	rtV173L, rtL180M	LAM and ETV (intermediate)
ETV + TDF	LIPA (4th month)	rtM204V (Wild type:N236)	rtV173L^e^, rtL180M (Wild type:L80, L180/A181)	LAM and ETV (intermediate)
ETV + TDF	Clonal analysis (5 clones) (4th month)	rtM204V (in clone 1-5)	rtV173L, rtL180M (in all clones)	LAM and ETV (intermediate

^a^ LAM – associated amino acids at position M552, L528 and V555 is previous nomenclature[20]

^b^ ETV intermediate: by L180M+M204V mutations; according to EASL Clinical Practice Guidelines 2009[1]

^c^ NT: Not tested

^d^ TDF intermediate: by N236T mutation; according to EASL Clinical Practice Guidelines 2009[1]

^e^ Detected after nested HBV PCR.

^f^ When band densities in LIPA assays are compared, wild-type probe band is denser than variant-type probe bands in this LIPA assay and of equal density in others LIPA assays.

**Table2:** Clonal analysis results of multidrug-resistant HBV strain in sera samples

**Clone**	**Sample**						**Mutation**						
		L80V	L91I	R120K	H126R	S135Y	V173L	L180M	A181V	M204I	L231V	Y245H	H248N
1	Sera, 11th month in ADV therapy		CTT → ATT	AGG → AAG	CAC → CGC	TCC → TAC							CAT¦AAT
2		TTA → GTA	CTT → ATT							ATG¦ATT		TAC¦CAC	
3		TTA → GTA									CTG¦GTG		
4									GCT¦GTT				
5		TTA → GTA	CTT → ATT							ATG¦ATT		TAC¦CAC	
6			CTT → ATT	AGG¦AAG	CAC¦CGC	TCC¦TAC							CAT¦AAT
		L80V	L91I	R120K	H126R	S135Y	V173L	L180M	A181V	M204V	L231V	Y245H	H248N
1	Sera, 4th month in ETV+TDF therapy		CTT → ATT	AGG¦AAG	CAC → CGC	TCC → TAC	GTG → TTG	CTG → ATG		ATG → GTG			CAT → AAT
2			CTT → ATT	AGG → AAG	CAC → CGC	TCC → TAC	GTG → TTG	CTG → ATG		ATG → GTG			CAT → AAT
3			CTT → ATT	AGG → AAG	CAC → CGC	TCC → TAC	GTG → TTG	CTG → ATG		ATG → GTG			CAT → AAT
4			CTT → ATT	AGG → AAG	CAC → CGC	TCC → TAC	GTG → TTG	CTG → ATG		ATG → GTG			CAT → AAT
5			CTT → ATT	AGG → AAG	CAC → CGC	TCC → TAC	GTG → TTG	CTG → ATG		ATG → GTG			CAT → AAT
